# Healthcare professionals' willingness to utilize a mobile health application for adverse drug reaction reporting in a limited resource setting: An input for digital health, 2023

**DOI:** 10.1016/j.eurox.2024.100324

**Published:** 2024-06-27

**Authors:** Abiy Tasew Dubale, Abiyu Abadi Tareke, Fikadu Wake Butta, Adamu Ambachew Shibabaw, Ermias Bekele Eniyew, Mohammedjud Hassen Ahmed, Sisay Yitayih Kassie, Addisalem Workie Demsash, Alex Ayenew Chereka, Geleta Nenko Dube, Agmasie Damtew Walle, Gemeda Wakgari Kitil

**Affiliations:** a Department of Health Informatics, College of Health Science, Mattu University, Mattu, Ethiopia; b Department of Midwifery, College of Health Science, Mattu University, Mattu, Ethiopia; c Department of Health Informatics, College of Health Science, Wallo University, Wallo, Ethiopia; d Deparment of Monitoring and Evaluation, West Gondar Zonal Health Department, Gondar, Ethiopia; e Department of Health Informatics, Asrat Woldeyes Health Science Campus, Debre Berhan University, Debre Berhan, Ethiopia

**Keywords:** MHealth application, Adverse drug reaction, Healthcare professionals, Ethiopia

## Abstract

**Background:**

Adverse drug reactions (ADRs) are a significant public health concern, particularly in limited resource settings where underreporting is prevalent due to various challenges. Mobile health applications (mHealth apps) offer a promising solution to enhance pharmacovigilance by facilitating easier and more efficient ADR reporting. However, despite the increasing availability and use of mHealth apps, there is a lack of evidence on healthcare professionals' willingness to adopt them for ADR reporting in resource-constrained environments. Therefore, this study aimed to assess the willingness of healthcare professionals in Ethiopia to utilize mobile health applications for adverse drug reaction reporting and identify associated factors.

**Methods:**

We carried out a cross-sectional study involving 422 healthcare professionals working in institutional settings. We gathered data through a pretested questionnaire that participants completed themselves. We inputted the data using Epi Data V.4.6 and analyzed it using SPSS V.26. Our analysis involved conducting multivariable logistic regression to identify the factors influencing the likelihood of healthcare professionals using mobile applications to report adverse drug reactions.

**Results:**

The study involved 389 healthcare professionals. Approximately 301 (77.4 %) of them expressed willingness to utilize mobile applications for reporting adverse drug reactions. The willingness to utilize mobile applications was significantly associated with the type of mobile phone (smart: AOR 3.56; 95 % CI 2.15–5.67), basic computer training (AOR 4.43; 95 % CI 2.27–8.64), mobile health-related training (AOR 1.96; 95 % CI 1.01–3.79), attitude (AOR 4.01; 95 % CI 2.19–7.35), perceived ease of use (AOR 2.91; 95 % CI 1.59–5.23), and perceived usefulness (AOR 2.10; 95 % CI 1.15–3.85).

**Conclusions:**

Overall, there was a high proportion of healthcare professionals willing to use mobile devices for reporting drug adverse reactions. Their willingness correlated with factors such as the type of mobile phone, perceived ease of use, attitude, training, and perceived usefulness of mobile applications. With the increasing use of smartphones, motivation among healthcare professionals is rising. Basic computer and mHealth-related training are crucial for enhancing the acceptability of such applications and should be incorporated into future implementations. Taking these factors into account could offer insights into the design and implementation of mobile applications for adverse drug reactions in Ethiopia.

## 1 Background

Globally, Adverse drug reactions major (ADR) cause of morbidity and mortality across all age groups, with a substantial number of hospital admissions, as well as economic costs burden on society and the healthcare systems [1], [2]. World Health Organization (WHO) defines an ADR as, a response that is noxious and unintended; which occurs at doses normally used in humans for prophylaxis, diagnosis, or the modification of physiological function [3], [4]. Adverse drug reactions are an important public health challenge and are widely underreported, which underestimates the risks of medicines and impedes actions to improve medication safety Approaches [5]. In post-marketing surveillance, spontaneous reporting of adverse drug reactions (ADRs) to the national regulatory agency has been the leading method for decades [6]. In developed countries, ADR-related hospitalization was observed at a rate of 6.3 %, while in developing countries, it stood at 5.5 %. Furthermore, the median proportions of preventable ADRs were 71.7 % in developed nations and 59.6 % in developing ones [7], [8]. Concerning potential risk factors for ADRs, developing countries have greater proportions. In Ethiopia, the risk of ADR-related hospitalization is emerging as a significant health concern, particularly with the rising number of patients presenting with various diseases. Historically, ADRs were predominantly reported by healthcare professionals (HCPs). However, there has been a shift in recent times towards an increased interest in ADR reports directly submitted by patients. This shift reflects a growing acknowledgment of the additional value that patient-reported ADRs bring to the table [6], [9].

World Health Organization (WHO) recently put forth a Global Strategy for Digital Health 2020–2025 with several countries having already achieved key milestones [10]. Digital technology in health includes various technologies like information and communication, mobile health, medical recording, and telemedicine [11]. Digitizing health systems is considered as the potential to improve healthcare services [12]. The perspective of the World Health Organization on eHealth, such as mobile health (mHealth), is that it represents medical and public health practices facilitated by mobile devices [13]. Evidence shows that mHealth applications are effective in improving the healthcare system such as for reporting, self-care, self-management, self-efficacy, and medication adherence as well as in improving health behaviors [14]. mHealth applications designed for ADR communication aimed at patients have primarily emphasized pharmacovigilance, complementing ADR reports from healthcare professionals to national medical authorities. While these applications may offer potential benefits, they also carry the potential to mitigate risks to patient safety. Healthcare professionals must be engaged from the outset of the conceptualization process, providing a solid foundation for interdisciplinary discourse surrounding mHealth applications [15]. Mobile health applications elevate the reporting of adverse drug reactions and enhance the communication of healthcare issues. The importance of these applications lies in their facilitation of two-way risk communication, which shortens the interval between experiencing and reporting an adverse drug reaction (ADR). This, in turn, can lead to greater knowledge among both patients and healthcare professionals [6]. Consequently, healthcare professionals assume a vital role in the pharmacovigilance system, necessitating extensive knowledge and expertise in medication safety. This is particularly crucial for the timely identification, detection, management, and reporting of adverse drug reactions (ADRs) [2], [16].

Various studies have pointed out that a significant proportion of adverse drug reactions go unreported by healthcare professionals, particularly in developing countries. This can be attributed to several factors, including a lack of awareness and knowledge about pharmacovigilance. It is estimated that adverse drug reaction reporting accounts for only 6 to 10 % of all ADR occurrences [2]. Therefore, healthcare professionals are expected to regard adverse drug reaction (ADR) reporting as a professional obligation. This is crucial because an effective system for ADR reporting plays a pivotal role in enhancing patient care and safety, thereby contributing to overall improvements in health outcomes.

The Ethiopian government has emphasized utilizing health information technology as a pivotal tool for advancing healthcare access and quality within the Ethiopian Health Sector Transformation Plan [17]. However, there is no available information in the literature about Willingness to utilize mobile health applications for adverse drug reaction reports among healthcare professionals. This study aimed to determine the Willingness to utilize mobile health applications for adverse drug reaction reports among healthcare professionals and the factors that influence their Willingness mobileplications.

## 2 Method of study

### 2.1 Study area and period

The research was conducted in the Ilu Abba Bor and Buno Bedelle Zones within the Oromia Regional State of southwest Ethiopia. Ilu Abba Bor Zone is situated approximately 600 km away from Addis Ababa, while Buno Bedelle Zone is around 483 km away from the capital. The study included five public hospitals: Bedele Hospital, Dembi Hospital, Darimu Hospital, Mettu Karl Comprehensive Specialized Hospital, and Chora Hospital.

Data collection took place over a period from January 20, 2023, to March 28, 2023, encompassing both zones and all five hospitals. These zones were selected due to their representation of varied healthcare settings within the region.

### 2.2 Study design

The study utilized an institution-based cross-sectional design.

### 2.3 Study population, sampling procedure, and sample size determination

The study population comprised all full-time healthcare practitioners working in the southwest Ethiopian zones of Buno Bedele and Illu Aba Bora. With the single population proportion formula, the sample size was calculated. Since the magnitude of willingness to utilize mobile applications for ADR is unknown, a 50 % proportion, a precision of 5 % and 95 % confidence interval, and a non-response rate of 10 % was taken to calculate the sample size. $\frac{n=\left(z_{2}^{\alpha }\right)2\;\times p\left(1-p\right)}{d2},n=\frac{1.96^{2}\times 0.5\left(1-0.5\right)}{\left(0.05^{2}\right)}\;=384.2$ then we consider a non-response rate of 10 %. Finally, with *n* representing the estimated sample size, *p* denoting the single population proportion we use (set at 50 %), and *Z*/2 indicating the 95 % level of confidence interval due to the unexplored willingness to utilize mobile applications for ADR in Ethiopia, and *d*2 standing for a 5 % margin of error, we calculate 384 + 384(0.1)= 422 A total of 422 healthcare professionals participated in this study, enrolled through a simple random sampling technique. Five fully functional hospitals and at the time of this study there were 1478 healthcare providers. However, our study included 1286 healthcare professionals because our study participants were physicians, nurses, and pharmacy professionals among those working in those hospitals were found in Illu Aba Bora and Bono Bedele Zones during the data collection period of the study. Initially, the total sample size was proportionally allocated based on the number of healthcare professionals in each hospital. Subsequently, health professionals were randomly selected from these hospitals.

### 2.4 Study variables and measurements

In this study, we focused on determining the willingness of individuals to use a mobile application for reporting adverse drug reactions (ADRs), and we also explored the factors associated with this willingness. To assess this, we took into account several predictor variables, including sociodemographic characteristics, perceived usefulness, patterns of mobile device usage, perceived ease of use, and overall attitude toward mobile applications.

For clarity, we defined willingness to utilize a mobile application as the likelihood of individuals using a specific computer program or health software installed on their mobile devices for reporting adverse drug reactions. Respondents were specifically asked if they would be willing to utilize these mobile applications for reporting ADRs to gauge their willingness. Finally, we measured the willingness to utilize these mobile applications by calculating the median scores obtained from respondents' answers [18].

If an individual's score was equal to or greater than the median, they were considered willing to use mobile health applications; otherwise, they were considered not willing to use them.

For the composite variables, we utilized a 5-item, 5-point Likert scale. The scale had five points for "strongly agree" and one for "strongly disagree." We totaled the scores of the elements inside each composite variable and divided them by the total number of items to create a composite variable scale (from 1 to 5) for data analysis. Lastly, we divided the composite variable score into two categories: "Yes" and "No." The study evaluated the attitudes of health professionals using a 5-point Likert scale. This scale included 10 items for one aspect and 5 items for another. Afterward, the Likert scale scores were divided into two groups. Respondents who scored at or above the median were considered to find the aspect useful or easy to use, while those scoring below the median were considered to find it not useful or not easy to use, respectively. Finally, the overall score was categorized as either "Yes" or "No" accordingly [18], [19].

### 2.5 Data collection tools and procedures

Data were collected utilizing standardized self-administered, pretested questionnaires adapted from diverse literature sources. The information was gathered through the employment of a structured English questionnaire. The questionnaire was adopted from previously published articles [15], [18], [20], [21], [22], [23], [24]. The dependent variable of the study was mobile health application for adverse drug reactions Independent variables include: socio-demographic characteristics (age, sex, marital status, religion, profession, year of experience, and education level), Mobile phone pattern (accessibility, type of mobile phone), organizational variable and attitudes on mobile health applications for adverse drug reactions were also addressed as independent variables. The questionnaire provides a succinct explanation of the research's purpose to participants. Participants were invited to volunteer during the study period, and those who consented were provided with the questionnaires. Before the actual data collection, the questionnaire was pre-tested. Data collectors received two days of training.

### 2.6 Data collection and quality control

Before the data collection date, both data collectors and supervisors underwent training covering the study's objectives, data collection procedures, tools for data collection, participant engagement techniques, ensuring data confidentiality, and respecting participants' rights. Subsequently, supervisors and the investigator verified the completeness of the questionnaires. Before the commencement of data collection, a pretesting phase involving health professionals (10 % of the total sample size) at Mettu Karl Comprehensive Specialized Hospital was conducted. This step ensured the completeness and consistency of the data. After the pretest, necessary modifications were made to the questionnaires based on the findings, before the official data collection began. The reliability was obtained by calculating the value of Cronbach’s Alpha values were all above (α = 0.84). The questionnaires were checked for missing values discrepancies, and completeness. Lastly, two days of training were provided to three onsite supervisors and four health informatics specialists who would be collecting the real data.

### 2.7 Data processing and analysis

Data entrance was conducted using Epi Data V.4.6, followed by exporting the data to SPSS V.26 for subsequent analysis. Before analysis, thorough data cleaning and cross-checking were performed. Descriptive statistics, including frequencies and percentages, were utilized and presented through graphs and tables. To analyze the association between the independent variable (Willingness to utilize mobile health apps for managing adverse drug reactions) and the dependent variable, binary logistic regression analysis was employed. Variables with a p-value below 0.25 in the bivariable analysis were incorporated into the multivariable analysis to address potential confounding factors. To determine whether multicollinearity existed among independent variables, the variance inflation factor (VIF) was used with a threshold of 10, and no evidence of it was discovered. Subsequently, the model fit was evaluated using the Hosmer and Lemeshow goodness-of-fit test, indicating a favorable fit for the data (p = 0.703). Variables deemed significant based on the adjusted Odds Ratio (AOR), with a 95 % confidence interval and p-value < 0.05, were considered determinant factors of user satisfaction.

### 2.8 Ethical consideration

The Institutional Review Board (IRB) at Mattu University, with reference number RPG/289, diligently reviewed and granted ethical clearance for our study. We collaborated closely with the zonal health bureau to obtain supporting letters, showcasing our commitment to ethical research practices. Following this, we meticulously obtained written consent from every study participant, a crucial step in ensuring the protection of their rights and privacy. Our data collection process was designed with utmost care, prioritizing anonymity and safeguarding the confidentiality of all participants throughout the study.

## 3 Results

### 3.1 Socio-demographic characteristics of respondents a

The study involved 387 participants, resulting in a response rate of 91.7 %. Among these participants, 231 (59.4 %) were male, and 236 (60.7 %) were aged between 20 and 29 years, with an average age of 31 ± 9.2 years. The largest professional group among the respondents was nurses, totaling 221 (56.8 %). Additionally, 91 individuals (23.5 %) held an educational degree. Moreover, the majority of respondents, comprising 247 (63.5 %), had four or fewer years of work experience (See Table 1).

**Table 1:** Socio-demographic characteristics of healthcare professionals working in a limited resource setting, 2023 (n = 389).

Variables	Category	Frequency	percent
age	20-30	236	60.7
	31-40	91	23.4
	> 40	60	15.4
sex	Male	158	40.6
	female	231	59.4
Marital status	Married	217	59.4
	Single	146	37.5
	Divorced	12	3.1
Religion	Orthodox	263	67.6
	Protestant	75	19.3
	Muslim	32	8.2
	others	19	4.9
profession	Nurse	221	56.8
	Physician	110	28.3
	Pharmacy	58	14.9
Year of experience	< =4	247	63.5
	5-9	93	23.9
	= >10	49	12.6
Educational status	diploma	125	23.4
	degree	91	32.1
	doctor	83	21.3
	MSc	90	23.1

### 3.2 Patterns of mobile phone use and organizational characteristics of

A total of 377 (96.9 %) respondents reported owning a mobile phone, with the vast majority (251, 66.6 %) owning smartphones. More than three-fourths of respondents (313, 80.5 %) had received basic computer training, while a smaller proportion (124, 31.9 %) had received mHealth-related training. Additionally, the majority of respondents (237, 60.9 %) reported having internet access through their organization. Of these, 254 (65.3 %) used the internet via their mobile phones, with only 37 (9.5 %) accessing it for health-related information (Table 2).

**Table 2:** Patterns of mobile phone use and organizational characteristics among healthcare professionals working in a limited resource setting, 2023(n = 389).

Variable	Category	Frequency	Percent
Do you have a phone?	Yes	377	96.9
	No	12	3.1
Mobile phone type	Smart	251	66.6
	Standard	126	33.4
Have you ever taken basic computer training	Yes	313	80.5
	No	76	19.5
mHealth related training	Yes	124	31.9
	No	265	68.1
Self-reported basic computer skills	Yes	246	63.2
	No	143	36.8
Access to the internet	Yes	237	60.9
	No	152	39.1
Use the internet via your mobile phone	Yes	254	65.3
	No	135	34.7
How often do you search for health-related information online?	several times a day or daily	107	27.5
	weekly or rarely	237	60.9
	Never	45	11.6
Use the internet to browse health-related data	Yes	348	89.5
	No	41	10.5
for what purpose do you use the internet via your mobile phone	To get health information	60	15.4
	To communicate with my friends	105	27.0
	To get daily news	94	24.2
	To manage patients’	68	17.5
	health data	37	9.5
	For reporting purpose	17	4.4
	Other	8	2.1
Is there management support to implement the eHealth system from your hospital	Yes	75	19.3
	No	314	80.7

### 3.3 Willingness to utilize mHealth applications for adverse drug reaction report

**Fig. 1:**
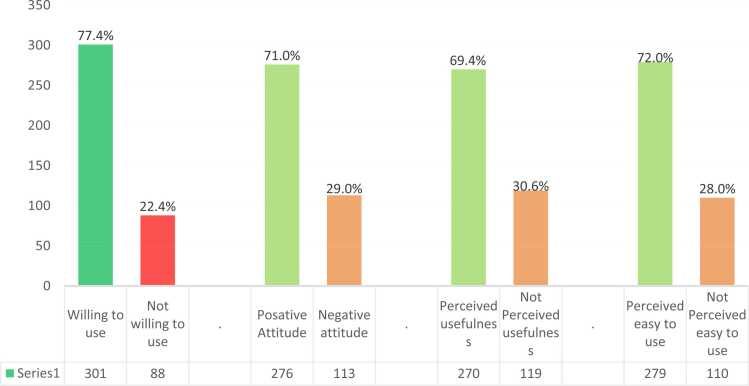
Willingness to utilize mobile health applications for adverse drug reaction reports among healthcare professionals working in a limited resource setting, 2023(n = 389).

Out of the total respondents, 301 healthcare professionals (77.4 %) expressed willingness to utilize mobile applications for reporting adverse drug reactions (ADRs). Additionally, 276 respondents (71.0 %) exhibited a positive attitude toward the use of such applications, and 279 (72.0 %) perceived them as easy to use for ADR reporting. Moreover, 270 respondents (69.4 %) acknowledged the usefulness of mobile applications in reporting adverse drug reactions (Fig. 1).

### 3.4 Factors associated with willingness to utilize the mHealth application for ADR

The outcomes of the bivariate analyses revealed that various factors such as age, gender, educational background, professional experience, type of mobile phone, computer proficiency, training in mHealth, attitude, internet accessibility, internet usage via mobile phones, perceived usefulness, and ease of use were correlated with the willingness to utilize mHealth apps for managing adverse drug reactions, with a significance level below p < 0.25. Subsequently, all these associated factors were incorporated into a multivariable logistic regression model. After adjusting for confounding variables, the multivariable logistic regression model highlighted the significance of mobile phone type, basic computer training, mHealth training, perceived usefulness, positive attitude, and ease of use in influencing the willingness to adopt mobile applications for managing adverse drug reactions.

Based on the results presented in the table below, individuals with smartphones were 3.56 times more inclined to utilize mobile applications for managing adverse drug reactions (AOR 3.56; 95 % CI 2.15–5.67) compared to those with standard phones. Moreover, respondents who underwent basic computer training exhibited a 4.43 times higher likelihood of using mobile health applications for adverse drug reactions (AOR 4.43; 95 % CI 2.27–8.64) compared to their counterparts, all other factors held constant. Additionally, respondents who received training specifically in mHealth were twice as likely to express willingness to utilize mobile health applications (AOR 1.96; 95 % CI 1.01–3.79) compared to those without such training.

Similarly, individuals who held a positive attitude towards mobile applications for managing adverse drug reactions were four times more inclined to utilize such applications (AOR 4.01; 95 % CI 2.19–7.35) compared to their counterparts. Likewise, respondents who perceived mHealth applications as easy to use were three times more likely to express willingness to utilize them (AOR 2.91; 95 % CI 1.59–5.23) compared to those who did not perceive them as easy. Similarly, individuals who perceived mobile applications as useful for managing adverse drug reactions were twice as likely to express willingness to utilize them (AOR 2.10; 95 % CI 1.15–3.85) compared to respondents who did not perceive them as useful (Table 3).

**Table 3:** Willingness to utilize mobile health application for adverse drug reaction report among healthcare professionals working in a limited resource setting, 2023(n = 389).

Character	category	willingness to utilize a Mobile App for ADR				
		Yes (%)	No (%)	COR (95 % CI)	AOR (95 % CI) p-value	
Age	20-30	186	50	0.58(0.31-1.08)	0.39(0.18-1.80)	0.022
	31-40	74	19	0.55(0.26-1.16)	0.574	0.226
	> 40	41	19	**1**	1	
sex	Female	125	33	1.18(0.72-1.93)	1.37(0.74-2.50)	0.310
	Male	176	55	**1**	1	
Educational status	diploma	82	43	1	1	
	degree	77	14	2.425(1.26-4.67)	1.02(0.42-2.46)	0.955
	doctor	68	15	0.841(0.38-1.84)	1.09(0.447-2.69)	0.841
	MSc	74	16	1.02(0.47-2.22)	1.05(0.427-2.62)	0.903
Year of experience	< =4	191	56	1	1	
	5-9	68	25	1.76(0.75-4.13)	1.73(0.88-3.42)	0.111
	= >10	42	7	2.20(0.89.5.55)	0.56(0.20-1.58)	0.273
Mobile phone type	smart	214	37	3.56(2.15-5.67)	**3.10(1.48-6.47)**	**0.003**
	standard	78	48	1	1	
basic computer training	Yes	259	54	4.09(2.39-7-01)	**4.43(2.27-8.64)**	**0.000**
	No	41	35	1	1	
mHealth related training	Yes	69	55	0.18(0.11-30)	**1.96(1.01-3.79)**	**0.045**
	No	232	33	1	1	
Access to the internet	Yes	202	35	3.18(1.95-5.18)	1.57(0.80-3.05)	0.182
	No	98	54	1.00		
Internet use via mobile phone	Yes	206	48	1.81(1.11-2.93)	0.40(0.18-1.94)	0.035
	No	95	40	1	1	
Attitude	positive	230	46	1.40(0.87-2.27)	**4.01(2.19-7.35)**	**0.000**
	Negative	71	42	1	1	
Perceived usefulness	Yes	219	51	1.93(1.18-3-17)	**2.10(1.15-3.85)**	**0.015**
	No	82	37	1	1	
Perceived easy to use	Yes	233	46	3.12(1.90-5.14)	**2.91(1.59-5.23)**	**0.001**
	No	68	42	1	1	

## 4 Discussion

Mobile device penetration provided a potential opportunity for using mHealth applications in low-income countries. Access and type of mobile phone technology are preconditions among other required infrastructures to enable the use of m-Health technology in health care delivery. The objective of this study is to evaluate the willingness to adopt mobile applications for managing adverse drug reactions, along with identifying the factors associated with this inclination. Our findings indicate that a substantial majority of respondents, 301 healthcare professionals (77.4 %), are willing to utilize mobile applications for reporting ADRs. This high level of willingness suggests a positive attitude towards integrating mobile technology into healthcare practices, which could be leveraged to enhance pharmacovigilance efforts in resource-limited settings. This result was lower than the use mobile health application study done in Iran [25]. The findings indicate a high level of willingness among healthcare professionals to utilize mobile health applications for reporting adverse drug reactions, standing at 77.4 %. This result was higher than the study in Germany (42 %) among general practitioners [15]. The variation observed could potentially stem from differences in sample size, the composition of study participants (such as the inclusion of only general practitioners in Germany), the duration of the study period, and sociodemographic disparities among healthcare professionals. Furthermore, this study pinpointed several influential factors, encompassing mobile phone type, perceived usefulness, computer-related training, health-related training, attitude, and perceived ease of use, all of which were linked to the inclination to utilize a mobile health application for managing adverse drug reactions. Specifically, the type of mobile phone emerged as a significant determinant of willingness to adopt mobile health applications.

Health professionals who had smartphones were 3.56 times more likely to be willing to use mobile applications for ADR (AOR 3.56; 95 %CI 2.15–5.67) than those who had standard phones. This study is consistent with a study of Northwest Ethiopia [22] and the study in Nigeria [24] and Florida [26]. Furthermore, healthcare professionals who had received basic computer training were 4.43 times more likely (AOR 4.43; 95 % CI 2.27–8.64) to express willingness to utilize mobile health applications for managing adverse drug reactions. Similarly, those who underwent mHealth-related training exhibited a two-fold increase in likelihood (AOR 2.00; 95 % CI 1.01–3.79) of being willing to adopt mobile health applications for adverse drug reactions. This study supported a study done in Ethiopia [22], Northwest Ethiopia [27], and a qualitative study in Uganda [20]. This could be attributed to the fact that training tends to enhance healthcare professionals' readiness to embrace various mobile technologies. Additionally, it's plausible that healthcare professionals who underwent mHealth training were more inclined to utilize mobile applications; as such training could have heightened their familiarity and comfort with this technology. This showed that mHealth and basic computer training could be directly associated with healthcare professionals' eagerness to use mobile applications. So, healthcare professionals, who are trained in mHealth as well as computers, think that they will encounter any issues using the mobile application.

Healthcare professionals who held a positive attitude towards utilizing mobile applications for managing adverse drug reactions were four times more likely to express willingness to utilize mHealth applications (AOR 4.01; 95 % CI 2.19–7.35) compared to their counterparts who did not share such positive attitudes. This result was consistent with a study conducted in northwest Ethiopia [22], a study conducted in a low-income country among health professionals [28], Saudi Arabia [21], and Northwest Ethiopia [29]. The possible reason is healthcare professionals who had positive attitudes towards mobile technology were more likely to use mobile applications for ADR reports because they have a positive understanding of the benefit of using digital technology (mobile technology). Similarly, attitude influences willingness to mHealth application. In addition to this, health professionals with positive attitudes towards the mHealth application could increase and result in higher intention to use the mHealth application.

Likewise, healthcare professionals who perceived mHealth applications as user-friendly were three times more inclined to express willingness to utilize mobile health applications (AOR 2.91; 95 % CI 1.59–5.23) compared to their counterparts who did not perceive them as easy to use. This study is in line with a study in the Netherlands [6], Ethiopia [28], and Florida [26]. Healthcare professionals' inclination to use mobile apps was bolstered by their perception of the future utility, ease, simplicity, brevity, clarity, and logic of such applications, ultimately enhancing the reporting of adverse drug reactions, particularly given the importance of time constraints in reporting practices. Put differently, if healthcare professionals perceive a mobile health application as cumbersome, they are less likely to engage with it. Conversely, if they believe the application will be beneficial, any usability challenges may be surmountable. Thus, the ease of use plays a pivotal role in increasing healthcare professionals' willingness to adopt mHealth applications.

Similarly, respondents who recognized the usefulness of mobile applications for adverse drug reactions were twice as likely to express willingness to utilize them (AOR 2.10; 95 % CI 1.15–3.85) compared to respondents who did not perceive the applications as useful. This result is consistent with a study conducted in Saudi Arabia [21], [30]. This underscores the importance of healthcare professionals' belief in the value of an application; without perceiving its usefulness, they are less likely to utilize it. Therefore, it becomes crucial to ensure that the developed application indeed enhances desired health outcomes.

### 4.1 Limitations of this study and future research

This study has several limitations. Firstly, it did not encompass health centers, clinics, and private health facilities, which may restrict the representativeness of the findings. Secondly, the exclusive use of quantitative approaches might affect the generalizability of the results. To enhance the generalizability of our findings, future research endeavors could consider incorporating all types of public health and private facilities. Additionally, supporting the quantitative findings with a qualitative study could offer deeper insights. Lastly, employing a health information technology acceptance model in future studies could provide a comprehensive understanding of the factors influencing healthcare professionals' acceptance and utilization of mobile health applications.

### 4.2 Conclusion

In conclusion, the study revealed a high willingness among healthcare professionals to embrace mobile health applications for managing adverse drug reactions. This willingness was correlated with factors such as the type of mobile phone, attitude, perceived ease of use, training, and perceived usefulness. With the widespread adoption of smartphones, healthcare professionals are increasingly motivated to utilize such applications. Basic computer skills and mHealth-related training were found to enhance the acceptability of these applications, suggesting their importance in future initiatives. Incorporating these insights into the design and implementation of mobile applications for adverse drug reactions in Ethiopia could lead to more effective interventions.

### 4.3 Recommendations

To increase the willingness of healthcare professionals to use mobile applications for reporting adverse drug reactions, several strategic recommendations can be implemented. First, providing financial incentives or subsidies to help professionals upgrade to smartphones can significantly enhance their ability to adopt these applications. Additionally, offering comprehensive digital literacy programs, including basic computer training and mobile health-related training, is crucial. These programs should be mandatory to ensure that all healthcare professionals are equipped with the necessary skills to effectively use mobile apps.

Furthermore, fostering a positive attitude towards technology is essential. This can be achieved by launching awareness campaigns and workshops that highlight the benefits of mobile app usage, featuring success stories and testimonials from peers. Involving healthcare professionals in the app design process is another critical step. By ensuring that the interface is user-friendly and easy to navigate, and by providing ongoing support and feedback mechanisms, the perceived ease of use of these applications can be greatly improved.

Lastly, it is important to communicate the practical benefits of using mobile applications for reporting adverse drug reactions. Emphasizing advantages such as increased efficiency, accuracy, and patient safety, supported by data and case studies, can help professionals understand the value of adopting these technologies. By implementing these strategies, healthcare organizations can significantly boost the adoption of mobile applications, leading to more accurate reporting and enhanced patient safety.

### 4.4 Implications of the findings for practice and policy

The findings of this study, have significant implications for both practice and policy:

#### 4.4.1 Enhanced adverse drug reaction reporting

The study reveals a positive willingness among healthcare professionals to adopt mobile health applications for adverse drug reaction reporting. Implementing these applications in healthcare settings can enhance the reporting of adverse drug reactions, thereby improving patient safety and healthcare outcomes.

#### 4.4.2 Improved efficiency in reporting

Mobile health applications can streamline adverse drug reaction reporting, making it more efficient and less time-consuming for healthcare professionals. This can lead to quicker identification and management of adverse drug reactions, reducing patient harm and enhancing overall healthcare delivery.

#### 4.4.3 Support for healthcare professionals

Providing training and support for healthcare professionals in the use of mobile health applications is crucial for their adoption. Policies should focus on integrating training programs into healthcare education and continuous professional development to ensure the effective utilization of digital health tools.

#### 4.4.4 Policy recommendations

Policymakers should consider developing guidelines and regulations to support the integration of mobile health applications into healthcare systems. This includes ensuring data security and privacy, standardizing reporting protocols, and incentivizing healthcare organizations to adopt digital health solutions.

#### 4.4.5 Addressing resource limitations

In resource-limited settings, mobile health applications offer a cost-effective solution for adverse drug reaction reporting. Policies should promote the use of digital health technologies as part of broader healthcare infrastructure improvements.

#### 4.4.6 Patient-centric care

Enhancing adverse drug reaction reporting through digital health technologies contributes to patient-centric care by improving patient safety and healthcare quality. Policies should prioritize patient outcomes and safety in the adoption and implementation of digital health solutions.

## Authors' contributions

All authors contributed equally to the conceptualization, study design, data collection, analysis, and interpretation of the study. They have agreed to be held accountable for all aspects of the work. Furthermore, they collectively contributed to the formulation of the paper and critically revised its core intellectual content.

## Consent for publication

Not applicable.

## Funding

This study was self-funded.

## CRediT authorship contribution statement

**Ermias Bekele eniyew:** Visualization, Validation, Supervision, Investigation, Data curation. **Mohammedjud Hassen Ahmed:** Writing – review & editing, Visualization, Supervision, Conceptualization. **Sisay Yitayih Kassie:** Writing – review & editing, Visualization, Supervision, Data curation. **Addisalem Workie Demsash:** Writing – review & editing, Visualization, Validation, Supervision, Data curation. **Adamu Ambachew Shibabaw:** Writing – review & editing, Visualization, Validation, Supervision, Data curation. **Gemeda Wakgari Kitil:** Writing – review & editing, Writing – original draft, Validation, Supervision, Methodology, Formal analysis, Data curation, Conceptualization. **Fikadu Wake Butta:** Writing – review & editing, Visualization, Validation, Supervision, Methodology, Data curation. **Alex Ayenew Chereka:** Writing – review & editing, Visualization, Supervision, Methodology, Data curation, Conceptualization. **Geleta Nenko Dube:** Writing – review & editing, Visualization, Supervision, Investigation, Data curation. **Agmasie Damtew Walle:** Writing – review & editing, Visualization, Validation, Supervision, Data curation. **Abiy Tasew Dubale:** Writing – review & editing, Writing – original draft, Visualization, Validation, Supervision, Software, Methodology, Investigation, Formal analysis, Data curation, Conceptualization. **Abiyu Abadi Tareke:** Writing – review & editing, Visualization, Validation, Supervision, Conceptualization.

## Declaration of Competing Interest

The authors declare that they have no competing monetary interests or personal relationships that could have influenced the work reported in this paper.

## Data Availability

Data will be available upon reasonable request from the corresponding author. As the study area is one of the surveillance sites in the country, we are unable to make the data publicly available. The data can only be accessed upon request because public access is restricted by Mattu University, College of Health Sciences, the institution that owns the data. Therefore, you can obtain the data and other supplementary information by contacting the surveillance site coordinator office at Mattu University, College of Health Sciences via email at [milkiasdugas@yahoo.com].
